# Global burden of complete hearing loss in people aged 70 and above: 1990–2023 and projections for 2040

**DOI:** 10.1016/j.bjorl.2026.101938

**Published:** 2026-11-03

**Authors:** Xiaoli Luo, Di Wu, Quanwei Ren, Yilin Xiang, Yanli Zhao

**Affiliations:** Chongqing Traditional Chinese Medicine Hospital, Department of Otolaryngology, Jiangbei District, China

**Keywords:** Global burden of disease, Complete hearing loss, 70-years, Prevalence, Years lived with disability.

## Abstract

**Objective:**

This study aimed to describe the global and regional trends in the Disease Burden (DB) of complete hearing loss among individuals aged ≥70-years from 1990 to 2023, and to project prevalence and Years Lived with Disability (YLD) rates through 2040, so as to inform prevention and control strategies for complete hearing loss in this aging population.

**Methods:**

Using data from the Global Burden of Disease (GBD) 2023 study, we used Prevalence (PR) and YLD rates to describe the burden of complete hearing loss in individuals aged ≥70 years from 1990 to 2023, stratified by Socio‑Demographic Index (SDI) region, GBD region, and sex. Analytical methods included join point regression, smoothing regression, frontier analysis, slope index of inequality, concentration index, and Autoregressive Integrated Moving Average (ARIMA) models.

**Results:**

The DB of complete hearing loss was highest in Sub‑Saharan Africa, High‑income North America, and High‑income Asia Pacific. From 1990 to 2023, both PR and YLD declined globally, with larger decreases in low‑to‑middle SDI regions than in high‑SDI regions. A positive DB‑SDI correlation was observed only in high‑SDI settings. Regional inequality in DB lessened over the study period. Except for high‑SDI regions, future DB trends through 2040 remain uncertain globally and across the other four SDI strata.

**Conclusion:**

Although the global burden of complete hearing loss has improved, regional disparities persist, indicating substantial room for further gains. More targeted interventions to improve access to diagnosis and treatment are urgently needed across regions.

## Highlights

• We calculated and analyzed the relevant situation of disease burden from 1990 to 2023.
• We predict the development trend of disease burden from 2024 to 2040.
• The disease burden of people aged 70 and above with complete hearing loss has improved.
• More targeted interventions are needed to improve diagnosis and treatment.

## Introduction

According to the World Health Organization (WHO), complete hearing loss in adults is defined as a pure‑tone hearing threshold of ≥95 dB HL at frequencies of 0.5, 1, 2, and 4 kHz in the better‑hearing ear. This condition is classified as the most severe category within the seven‑level severity classification of the Global Burden of Disease (GBD).^1^ Complete hearing loss adversely affects communication, social interaction, emotional well‑being, and cognitive function in older adults.^2^ Individuals with complete hearing loss often struggle to sustain social connections and participate in daily activities, which may result in psychological sequelae including isolation, depression, and anxiety.^3^, ^4^, ^5^ Furthermore, complete hearing loss is associated with a higher risk of dementia and other cognitive impairments.^6^

In the United States, approximately half of adults aged ≥70-years have some degree of hearing impairment.^7^ The annual global financial burden of hearing loss is estimated by the WHO to be close to US$ 1 trillion.^8^ Although complete hearing loss affects a small proportion of the total population, it incurs substantial individual medical costs.

Globally, the burden of hearing loss is unequally distributed.^8^^,^^9^ Low‑ and middle‑income countries facing disproportionately greater challenges due to weaker health systems, shortages of trained personnel, and limited public awareness.^8^

Age 70 represents a pivotal threshold in complete hearing loss prevalence. In 2023, the global prevalence of complete hearing loss among individuals aged ≥70 years was 6.5 times that in those younger than 70, according to GBD data. This age also approximates the close of the window during which hearing interventions confer substantial cognitive benefits. A well‑known study in JAMA Neurology reported that hearing aid use reduced dementia risk by 61% in hearing‑impaired adults aged <70 years, whereas interventions initiated after age 70 offered only modest gains.^10^ In older adults, complete hearing loss arises largely from cumulative age‑related degeneration ‒ including hair cell loss and spiral ganglion neuron deterioration‑leading to a progressive, predominantly high‑frequency, and generally irreversible hearing loss.^11^ Its pathogenesis differs fundamentally from that observed in younger populations, in whom etiologies such as congenital anomalies, infectious causes, and genetic disorders are more prevalent.^12^

Previous GBD studies on hearing loss have largely focused on all severity levels combined or on age‑related hearing loss in the general population, often using a threshold of ≥60 years. To date, however, no study has specifically examined the global burden of complete hearing loss ‒ the most severe and disabling end of the hearing spectrum, with a strict focus on adults aged ≥ 70 years ‒ a group that carries the heaviest burden yet remains the least studied. Furthermore, frontier analysis has only recently been applied to hearing loss research, and our study is among the first to use it in the context of complete hearing loss in older adults. In this study, we present the first comprehensive analysis of DB in the population aged ≥70 years, integrating frontier analysis, projections through 2040, SDI‑based inequality assessment, and post‑pandemic trend surveillance within a single analytical framework. This multidimensional approach moves beyond a purely descriptive account of historical trends, allowing us to identify policy priorities and guide more targeted resource allocation.

## Methods

### Overview

The GBD 2023 study provided a systematic assessment of disease burden for 375 conditions and 88 risk factors across 204 countries and territories, along with estimates of healthy life expectancy.^13^ Data were drawn from a wide range of sources, including censuses, household surveys, civil registration and vital statistics, disease registries, health facility records, environmental monitoring, and satellite‑based observations.

The Annual Percentage Change (APC) measures the year‑on‑year change in disease burden indicators, while the Average Annual Percentage Change (AAPC) reflects the average rate over a specified period. The Estimated Annual Percentage Change (EAPC) was used to summarize the average annual rate of change over the study period, serving as a summary measure of long‑term trends. Join-point regression, the statistical method used in the GBD study,^13^ was employed to calculate the EAPC.^14^ A positive EAPC with a 95% Confidence Interval (95% CI) entirely above zero indicates a significant increase in burden over time; conversely, a negative EAPC with the upper bound of the CI below zero a significant decline. When the 95% CI contains zero, the trend is considered stable. The SDI is a composite measure of development, ranging from 0 to 1, derived from three components: total fertility rate among women under 25-years of age, average years of education among adults aged ≥15-years, and per capita income. Higher SDI values correspond to higher levels of socio‑demographic development.^15^

### Data sources

Data for this study were obtained from the Global Health Data Exchange (GHDx) platform (https://ghdx.healthdata.org/). Demographic and SDI data were retrieved from the same source. We extracted and analyzed prevalence, Years Lived with Disability (YLD), SDI, and population counts for individuals aged 70 years and older with complete hearing loss, covering the period from 1990 to 2023. This analysis encompassed global data, five SDI regions, 21 GBD regions, and 204 countries and territories. According to the GBD classification, complete hearing loss is defined as an average pure‑tone hearing threshold of ≥95.0 dB HL in the better ear, a definition consistent with the WHO grading system for hearing loss.^1^^,^^16^

### Analysis indicators

PR and YLD were selected as analytical indicators. PR is the proportion of individuals affected by a disease during a specified period. YLD measures healthy years lost to disability and reflects the associated health burden. Age‑standardized YLD were used to account for differences in population age structure.^17^ In the GBD framework, YLD are derived by multiplying the prevalence of each sequela by its disability weight, adjusting for comorbidity, and aggregating them to the cause level.^18^ Disability weights range from 0 (full health) to 1 (death‑equivalent) and are assumed to be consistent across regions.^19^ Because we focused solely on complete hearing loss, confounding by severity‑related variation in weights was avoided. All rates are per 100,000 population.

### Statistical methods

EAPC was calculated using joinpoint regression as 100 × (exp(β) − 1), where β is the regression coefficient from the model ln(value) = α + β × year + ε.^14^^,^^20^

We calculated the AAPC for prevalence and YLD using joinpoint regression, with the log‑transformed rate as the dependent variable and year as the independent variable. The AAPC was derived as a weighted average of segment‑specific APCs, allowing us to identify breakpoints, quantify the rate of change, and characterize temporal trends. Smoothing regression was used to evaluate the association of prevalence and YLD with SDI across regions.

Frontier analysis was used to explore the potential for DB reduction, accounting for regional differences in SDI.^21^ This method establishes a frontier representing the lowest achievable PR and YLD rates for each SDI level, identifying regions where actual DB exceeds the minimum achievable level and lags behind developmental expectations. These findings reveal opportunities for improvement and guide optimization of related strategies.

Inequality in disease burden was assessed using the slope index of inequality and the concentration index across 21 GBD regions and globally. A weighted regression model was used to correct for heteroscedasticity. The slope index was derived from sex‑specific prevalence, YLD, and SDI data from 1990 to 2023. The concentration index was calculated as the area under the Lorenz curve, fitted from the cumulative population distribution ranked by SDI and the corresponding prevalence and YLD.

We used the ARIMA model to project trends in prevalence and YLD globally and in five SDI regions from 2024 to 2040. The autoregressive (p) and moving average (q) orders were restricted to 0 or 1. Candidate models included ARIMA(1,1,0), ARIMA(0,1,1), and ARIMA(1,1,1), with and without a drift term; models automatically selected via the auto.arima function were also considered. The final model was selected based on the minimum corrected Akaike Information Criterion (AICc). To determine whether the series had a deterministic trend that could be captured by the drift term, we performed a *t*‑test on the drift coefficient in the ARIMA model.

All statistical analyses were performed using R software (version 4.5.2).

## Results

### Distribution of prevalence and YLD in 2023

In 2023, the global prevalence of complete hearing loss was 777.86 per 100,000 (95% CI: 561.67∼1034.3), and the age‑standardized YLD was 193.76 (95% CI: 112.46∼277.61). The prevalence and YLD across the five SDI regions are summarized in Table 1. In every SDI stratum, the rates were higher in women than in men. The low‑SDI region carried the heaviest burden, with a prevalence of 970.23 and a YLD rate of 237.76 (Fig. 1; Supplementary Table S1).

**Table 1:** PR and YLD rates in 1990 and 2023 by region and SDI stratum.

Measure	Location	1990		2023	
		Rate	95% CI	Rate	95% CI
PR	Global	828.98	595.53～1123.60	777.86	561.67～1034.30
YLD	Global	206.72	127.34～326.44	193.76	112.46～277.61
PR	Low SDI	1255.76	887.54～1719.72	970.23	685.77～1309.31
YLD	Low SDI	304.56	177.60～430.20	237.76	139.65～340.82
PR	Low‑middle SDI	1038.93	727.23～1413.12	791.91	558.15～1068.44
YLD	Low‑middle SDI	251.5	149.15～358.68	193.98	114.19～276.90
PR	Middle SDI	947.9	668.17～1291.63	760.53	541.18～1019.09
YLD	Middle SDI	231.54	135.52～331.94	187.78	108.98～266.49
PR	High‑middle SDI	875.04	610.87～1205.34	726.17	520.78～962.86
YLD	High‑middle SDI	214.79	126.16～310.05	179.89	105.10～255.45
PR	High SDI	847.81	601.13～1134.16	770.66	559.68～1017.23
YLD	High SDI	212.63	121.70～311.57	193.72	111.76～279.15
PR	Andean Latin America	552.42	393.07～770.21	641.39	449.82～865.22
YLD	Andean Latin America	137.21	81.07～211.44	159.76	92.67～231.62
PR	Australasia	870.44	661.59～1151.29	881.67	627.62～1158.81
YLD	Australasia	220.48	138.15～339.82	222.04	128.07～319.16
PR	Caribbean	485.62	341.73～680.82	527.68	375.98～717.16
YLD	Caribbean	121.41	72.32～192.94	131.14	75.23～190.02
PR	Central Asia	820.44	601.36～1121.52	769.04	538.37～1028.24
YLD	Central Asia	204.44	125.50～326.27	190.75	109.86～271.06
PR	Central Europe	766.77	547.25～1054.09	729.74	512.00～979.65
YLD	Central Europe	193.46	118.34～308.29	183.79	105.93～260.64
PR	Central Latin America	533.81	377.65～744.92	543.37	387.06～730.01
YLD	Central Latin America	132.66	79.04～211.52	134.64	78.15～192.04
PR	Central Sub‑Saharan Africa	1043.28	729.16～1458.86	1142.48	809.98～1545.79
YLD	Central Sub‑Saharan Africa	259.62	151.66～402.57	282.78	163.38～399.26
PR	East Asia	790.56	560.42～1096.01	774.98	557.37～1027.34
YLD	East Asia	197.05	117.63～308.10	192.74	113.62～271.28
PR	Eastern Europe	782.8	562.40～1068.79	737.85	519.94～987.35
YLD	Eastern Europe	196.19	120.88～308.78	183.62	105.41～262.35
PR	Eastern Sub‑Saharan Africa	1431.75	997.70～1970.94	1150.4	831.91～1524.44
YLD	Eastern Sub‑Saharan Africa	352.44	207.58～545.85	282.81	165.77～405.04
PR	High‑income Asia Pacific	738.83	524.37～1022.37	936.42	673.01～1228.46
YLD	High‑income Asia Pacific	188.08	113.31～300.11	237.11	137.98～344.32
PR	High‑income North America	1114.83	812.81～1527.53	1000.17	716.33～1338.15
YLD	High‑income North America	282.46	169.95～447.34	252.38	144.01～365.64
PR	North Africa and Middle East	1444.64	1069.14～1929.08	909.84	659.78～1216.01
YLD	North Africa and Middle East	356.81	214.78～555.99	226.24	131.62～326.06
PR	Oceania	702.38	492.39～1001.44	773.39	532.04～1070.10
YLD	Oceania	174.22	101.30～279.25	192.27	107.67～279.06
PR	South Asia	952.53	688.06～1288.05	736.57	515.18～999.91
YLD	South Asia	229.18	141.47～356.47	179.86	106.18～260.80
PR	Southeast Asia	848.39	619.00～1153.34	743.5	531.65～998.68
YLD	Southeast Asia	208.96	127.94～329.02	183.15	106.29～260.88
PR	Southern Latin America	822.26	581.55～1129.33	897.91	643.66～1207.29
YLD	Southern Latin America	207.89	127.00～329.18	226.43	129.39～330.96
PR	Southern Sub‑Saharan Africa	797.72	562.02～1098.39	893.43	627.13～1216.39
YLD	Southern Sub‑Saharan Africa	198.99	117.44～314.23	221.8	128.33～327.20
PR	Tropical Latin America	387.3	279.50～534.63	351.91	253.63～469.81
YLD	Tropical Latin America	95.95	57.87～152.16	87.11	50.06～123.94
PR	Western Europe	560.76	401.98～777.19	600.71	433.74～801.26
YLD	Western Europe	142.28	86.27～225.99	152.24	87.96～224.30
PR	Western Sub‑Saharan Africa	1179.72	846.35～1585.26	1392.12	990.72～1847.63
YLD	Western Sub‑Saharan Africa	291.32	179.82～450.36	341.3	199.95～489.61

**Fig. 1:**
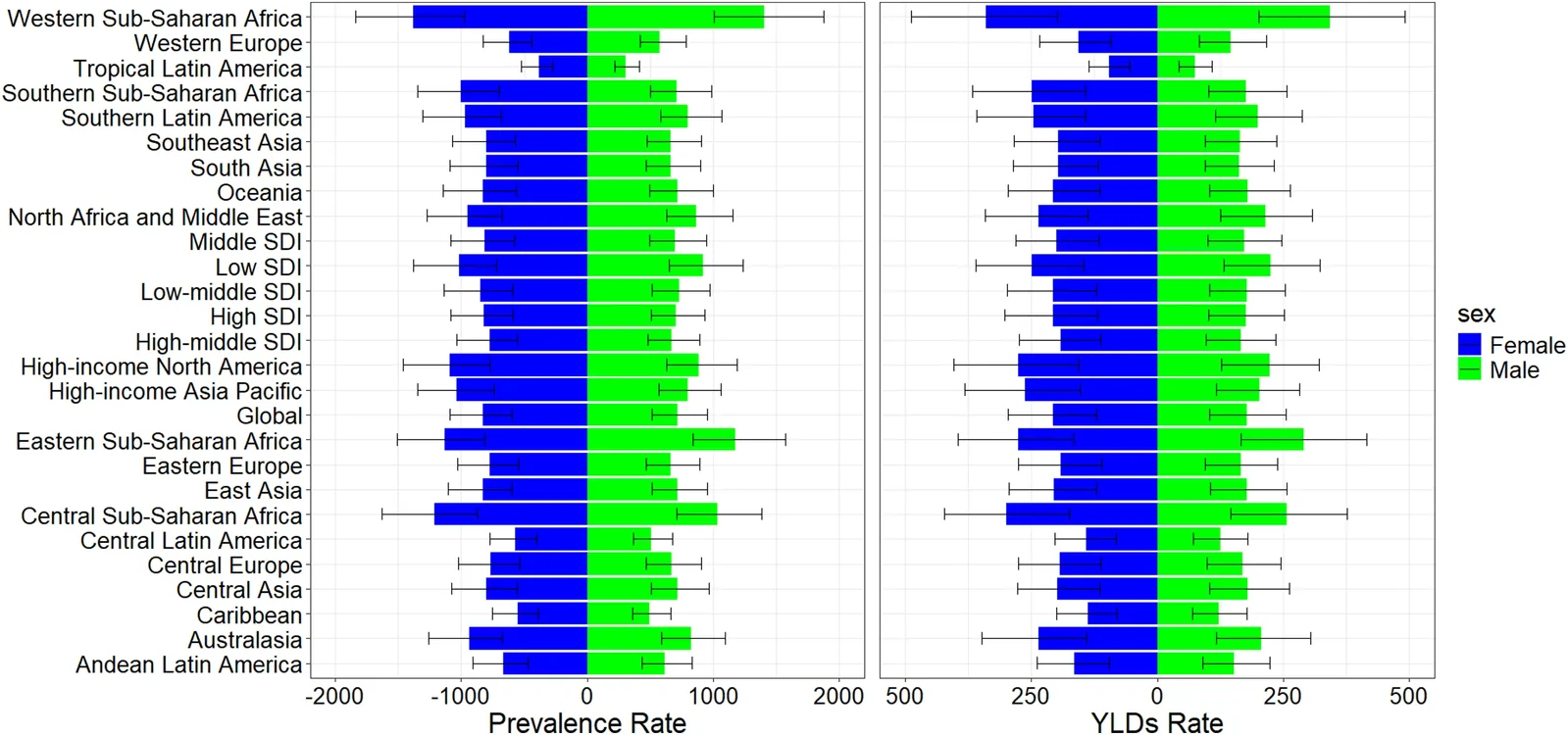
Prevalence rate and YLDs rate of 2023 by sex. Trends in PR and YLD from 1990 to 2023.

Tropical Latin America had the lowest prevalence in both sexes (305.53 in males and 386.02 in females), whereas Western Sub‑Saharan Africa had the highest (1402.17 and 1383.22, respectively). YLD followed a similar distribution, ranging from 75.77 (males) and 95.45 (females) at the low end to 342.40 and 340.33 at the high end (Supplementary Table S1). Sub‑Saharan Africa, High‑income North America, and the Asia‑Pacific region carried the heaviest overall burden.

At the country level, 155 countries had a PR between 500 and 1000 per 100,000, and 38 countries had a PR between 1000 and 2000. Meanwhile, 158 countries had YLD between 120 and 250, and 32 countries had rates between 250 and 350.

The optimal Joinpoint indicated that, compared with 1990, global PR and YLD rates declined significantly by 2023 across both sexes and all SDI regions (Fig. 2). The global AAPC from 1990 to 2023 was −0.17% for PR and −0.18% for YLD; for males, the corresponding values were −0.23% and −0.23%; for females, −0.12% and −0.20%, respectively (Supplementary Table S2). Across SDI strata, the AAPC for PR was −0.79% (low), −0.84% (low‑middle), −0.66% (middle), −0.57% (high‑middle), and −0.30% (high); the YLD AAPCs followed the same order: −0.76%, −0.80%, −0.63%, −0.54%, and −0.29%, respectively (Supplementary Table S2). Notably, from 2021 to 2023, global YLD rates rose significantly in both sexes.

**Fig. 2:**
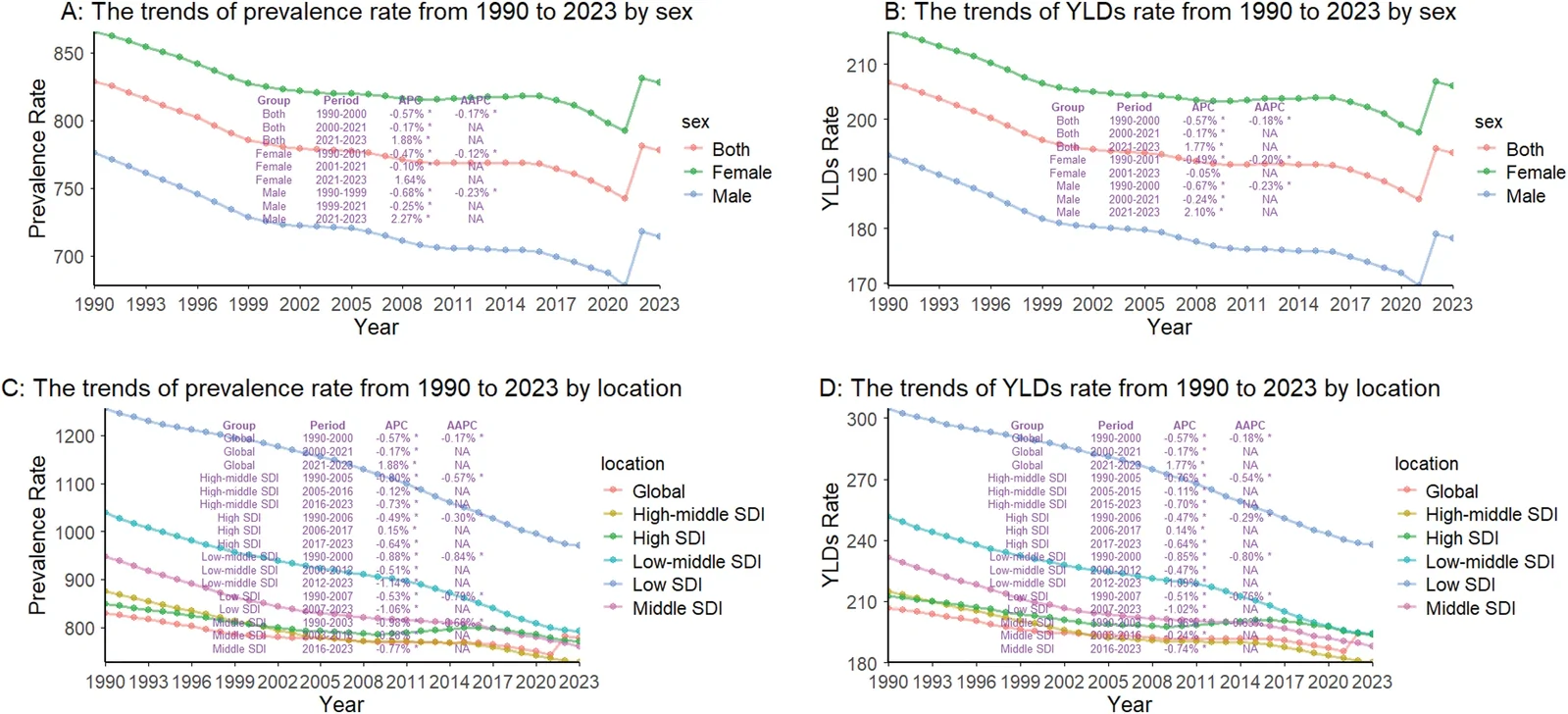
The trends of change on prevalence rate YLDs rate from 1990 to 2023.

From 1990 to 2023, global PR and YLD trends fell into three phases: 1990–2000, 2000–2021, and 2021–2023. The APC for PR was −0.57%, −0.17%, and 1.88% across these phases, and for YLD it was −0.57%, −0.17%, and 1.77%. The YLD APC was significant only in 1990–2000 and 2021–2023 (Fig. 1; Supplementary Table S2).

For males, the PR and YLD trends showed three phases (1990–1999/1990–2000, 1999–2021/2000–2021, and 2021–2023), with APCs of −0.69%/−0.67%, −0.25%/−0.24%, and 2.27%/2.10% (all significant). For females, the phases differed: 1990–2001 for both indicators, 2001–2021 for PR only, and 2021–2023 for PR versus 2001–2023 for YLD, with corresponding APCs of −0.47%/−0.49%, −0.10%/NA, and 1.64%/−0.05%. All sex and SDI-specific APCs and AAPCs were statistically significant, except for the male PR APC in 2021–2023 and the male YLD APC in 2001–2023. Notably, global PR and YLD rose sharply in 2021–2023 across both sexes, reversing the earlier declining trends (Fig. 2; Supplementary Table S2).

At the national level, the five countries with the largest prevalence reductions from 1990 to 2023 were the United Arab Emirates (−40.4%), Oman (−37.7%), Iraq (−37.6%), Saudi Arabia (−36.5%), and Kuwait (−35.8%). Similarly, the top five countries for YLD decline were the United Arab Emirates (−39.5%), Iraq (−36.5%), Oman (−35.8%), Saudi Arabia (−35.4%), and Kuwait (−35.1%). In contrast, prevalence increased in Andorra (5.0%), the Democratic People's Republic of Korea (1.7%), San Marino (1.6%), Japan (0.8%), and Australia (0.8%); YLD increased in Andorra (4.5%), San Marino (1.8%), the Democratic People's Republic of Korea (1.6%), and Japan (0.9%) (Fig. 3; Supplementary Table S3). However, these countries account for only a small share of the global population and their respective SDI regions; therefore, they barely influence the overall trends.

**Fig. 3:**
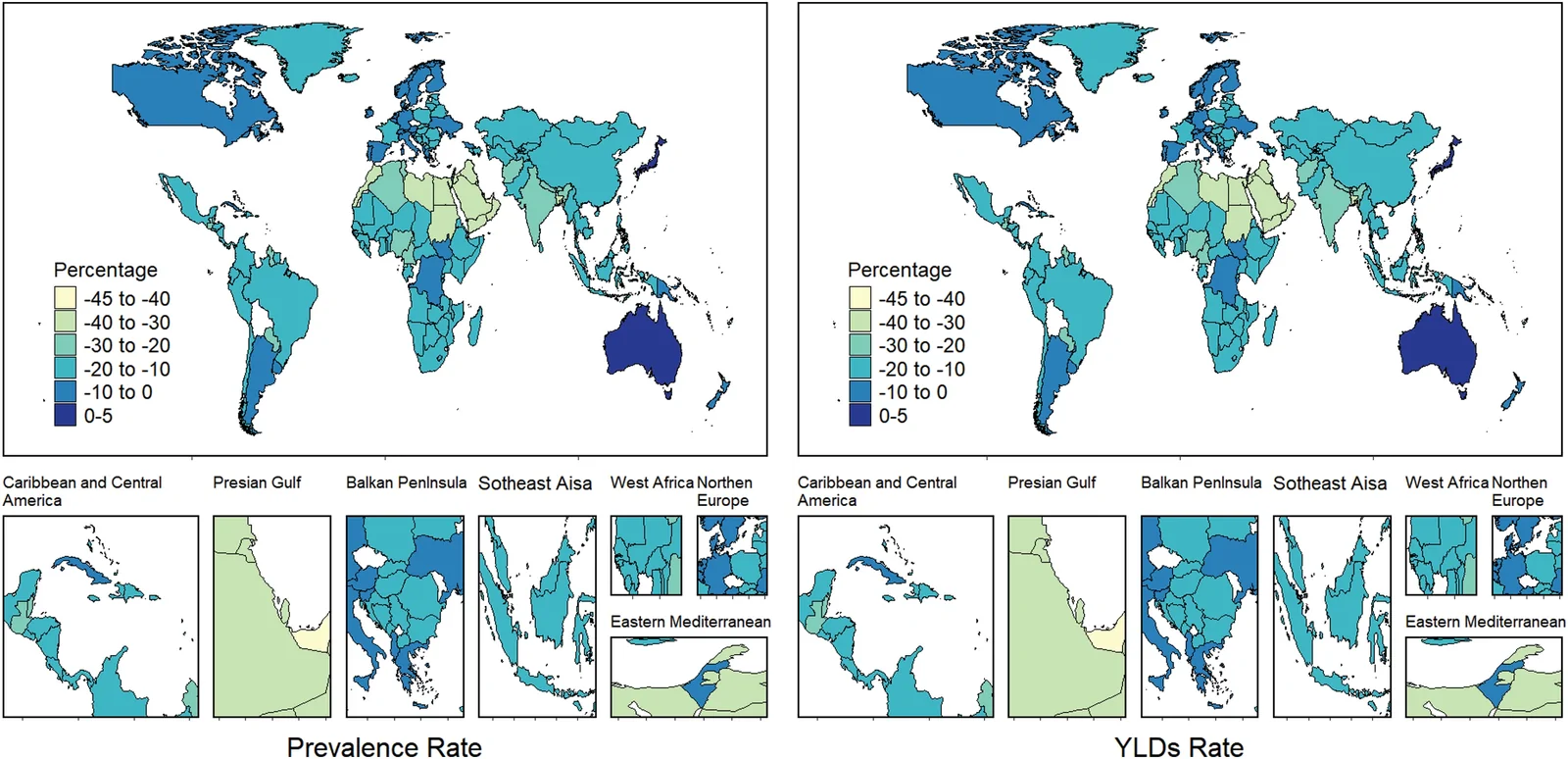
Change percentage of prevalence rate and YLDs rate of 204 countries from 1990 to 2023. Relationship between DB and SDI.

From 1990 to 2023, both PR and YLD were negatively correlated with SDI, albeit weakly (*r* = −0.428 and −0.393, respectively; p < 0.05). The correlations varied by SDI level: below 0.6, moderate negative correlations (*r* = −0.639 and −0.628, p < 0.05); above 0.6, weak positive correlations (*r* = 0.255 and 0.280, p < 0.05). Regional patterns also differed: positive in East Asia and high‑SDI regions and negative elsewhere (Fig. 4; Supplementary Table S4).

**Fig. 4:**
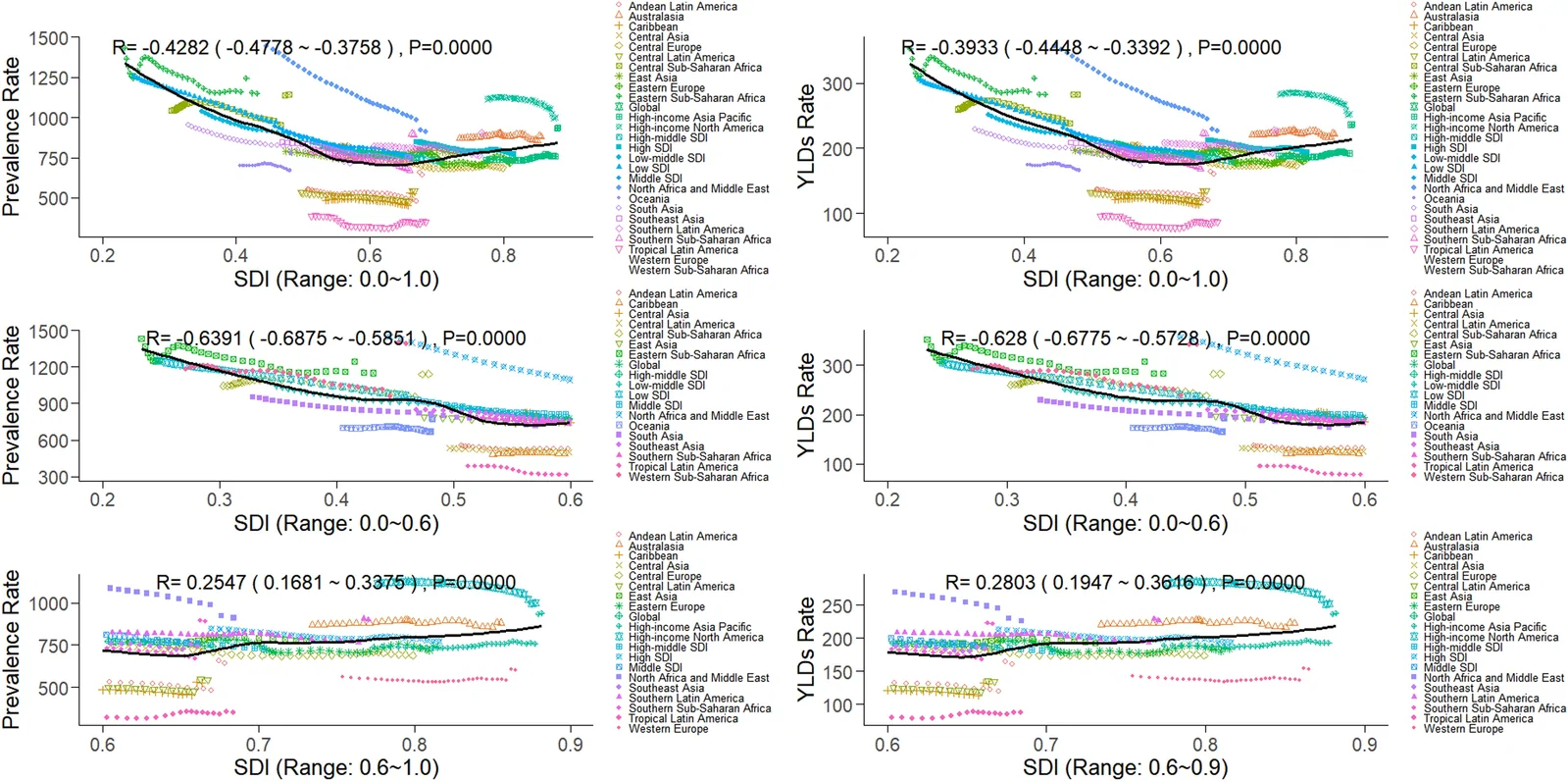
The correlation between prevalence rate, YLDs rate and SDI.

### Differences between disease burden improvement and ideal conditions

Frontier analysis established optimal PR and YLD benchmarks for each SDI level and quantified deviations. The frontier represents the minimum achievable rate at each SDI level. Gaps between actual rates and the frontier reflect unrealized health gains, indicating potential for DB improvement.

Fig. 5 displays the 2023 best‑practice frontier (top black line, panels A–D), which marks the maximum DB improvement achievable at a given SDI level. At lower SDI levels, the frontier declined as SDI increases; at higher levels, it remained relatively flat. In panels A and C, the blue dotted line tracks actual PR and YLD for each region from 1990 to 2023; the gap to the frontier reflects unrealized gains over that period, with most regions and years showing some shortfall. Panels B and D plot each region’s 2023 PR or YLD (blue for increases from 1990, orange for decreases). The distance from each dot to the frontier reflects regional unrealized gains in 2023. The seven black‑labeled regions‑Western Sub‑Saharan Africa, High‑income North America, High‑income Asia Pacific, North Africa and the Middle East, Southern Latin America, Southern Sub‑Saharan Africa, and Australasia-show the largest gaps and thus the greatest improvement potential. Blue‑labeled regions rank next, and red‑labeled regions rank last (Fig. 5; Supplementary Table S5).

**Fig. 5:**
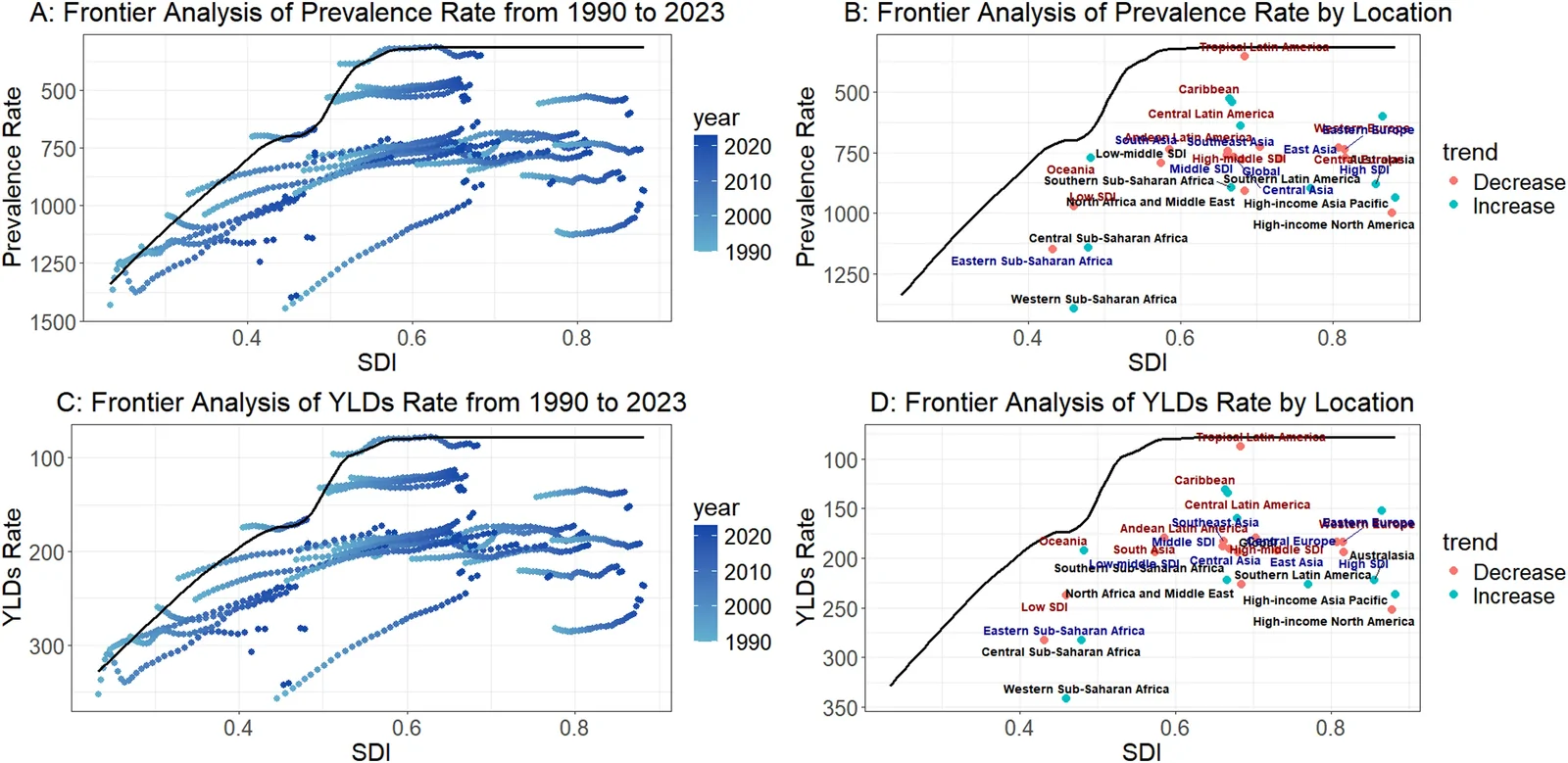
Frontier analysis on the relation between prevalence rate, YLDs rate and SDI. Regional inequality in PR and YLD, 1990–2023.

Inequality analysis revealed that PR and YLD were slightly negatively correlated with SDI in 1990, yet this correlation had nearly disappeared by 2023. No obvious sex differences in PR and YLD were found across SDI regions (Fig. 6). Although the inequality of DB declined moderately in 2023 compared with 1990, the improvement remained limited.

**Fig. 6:**
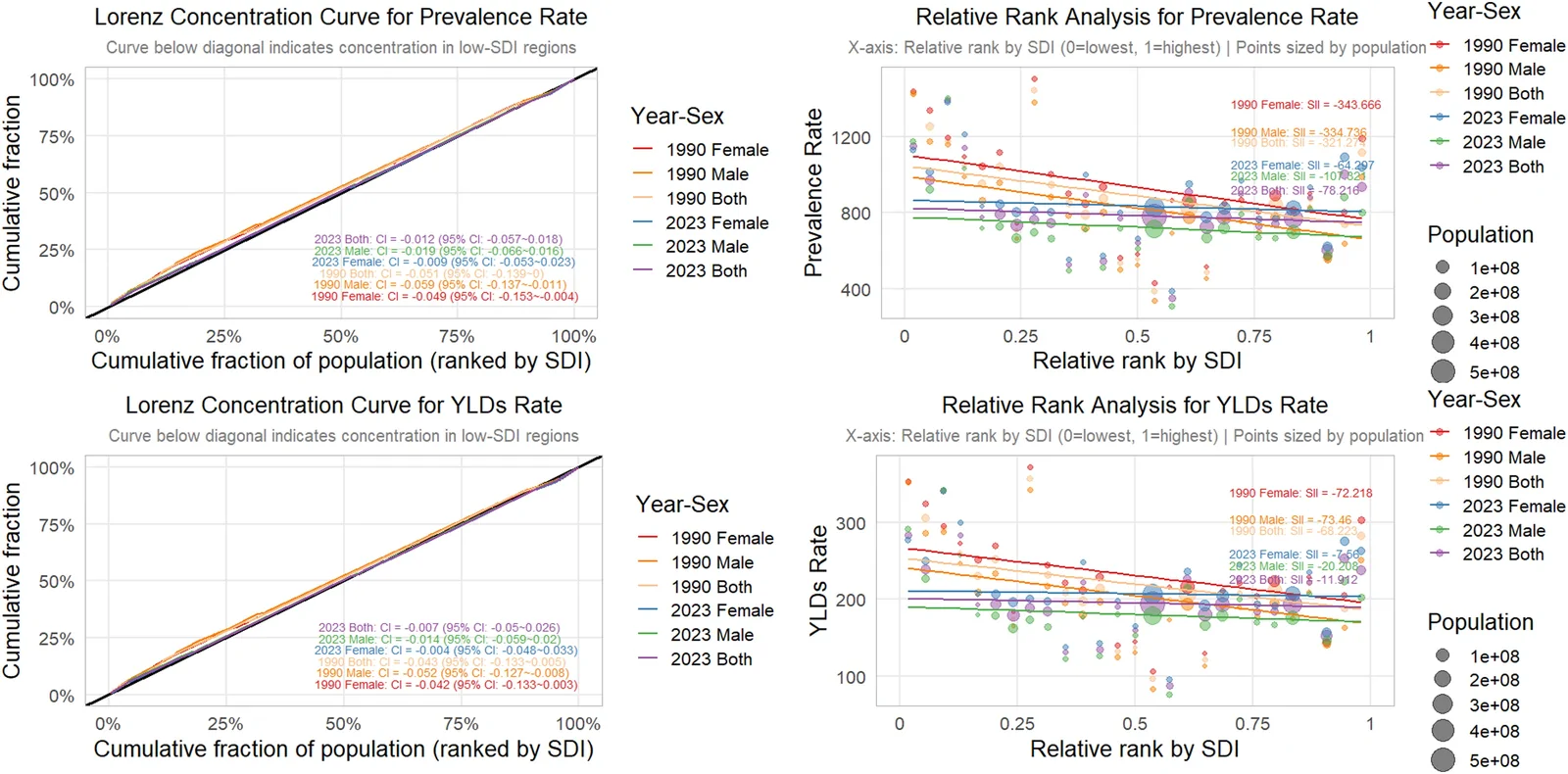
Inaquality analysis for prevalence rate and YLDs rate by sex year.

The ARIMA model was used to predict DB from 2024 to 2040. ARIMA(1,1,0) with drift was identified as the optimal model for high‑SDI regions, incorporating a first‑order autoregressive term and a first‑order difference to capture short‑term dependence within the time series. PR and YLD rates in this region are projected to show a slight declining trend, decreasing from 770.66 and 193.72 in 2023 to 730.36 and 184.02 in 2040. The AR(1) coefficients from ARIMA(1,1,0) with drift were 0.7805 and 0.7723, respectively (both p < 0.001). The drift coefficients were −2.3379 and −0.5730, respectively (both p < 0.001). The Ljung‑Box test indicated that residuals were consistent with white noise (p = 0.3031 and 0.2883, respectively). ARIMA(0,1,0), the pure random walk model, served as the optimal model for the global level and the other four SDI regions. Predicted values for 2024–2040 remained consistent with the 2023 observed data. The expanding prediction intervals suggested progressive accumulation of inherent uncertainty over time (Fig. 7, Supplementary Tables S1 and S6). Residuals of all final models passed the Ljung‑Box white noise test (p > 0.05), confirming adequate information extraction from the original datasets. We fitted an ARIMA(0,1,0) model with a drift term and tested the drift coefficient. Except for the high‑SDI region, indicating non‑significance and failure to reject the null hypothesis of zero drift. Therefore, we adopted a drift‑free random walk model for these strata (Fig. 7; Supplementary Tables S7).

**Fig. 7:**
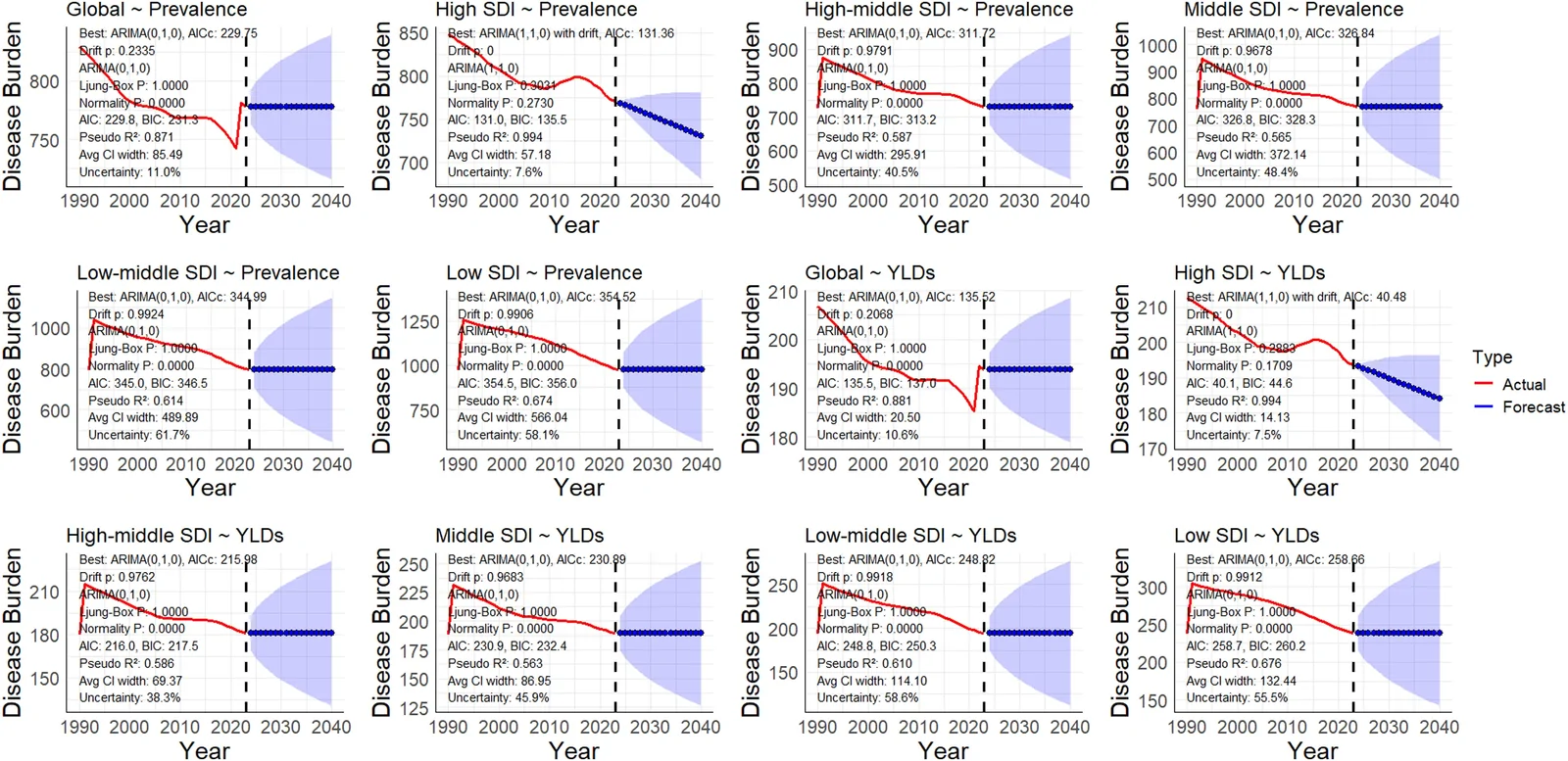
Trend forecast of prevalence rate and YLDs rate by 2040.

## Discussion

Compared with 1990, global PR and YLD of complete hearing loss in individuals aged ≥ 70 years declined significantly by 2023, with decreases observed in both sexes and across all SDI regions. This decline can be attributed to a slower increase in the numerator than in the denominator: according to GBD data, the population aged ≥ 70-years grew by 1.8 times, whereas the number of affected cases increased by only 1.5 times. The most notable improvements were observed in low‑SDI and low‑middle SDI regions, suggesting that older adults with age‑related hearing loss in these areas have already benefited from efforts to prevent or mitigate hearing loss. For example, over the past three decades, targeted initiatives in Western Sub‑Saharan Africa have substantially reduced hearing loss.^22^ Similarly, the national deafness prevention program launched by the Indian government covers 595 administrative districts, benefiting nearly 80% of the population, with service coverage expanding annually.^23^

PR and YLD rebounded from 2021 to 2023, coinciding with the COVID‑19 pandemic. As previous studies have not identified an elevated risk of SARS‑CoV‑2 infection or vaccination among individuals with hearing loss,^24^ this rebound is unlikely to stem from the virus itself. Instead, it may be attributable to two factors. First, expanded surveillance coverage in GBD 2023 captured previously unreported cases. Second, pandemic‑driven systemic disruptions to hearing‑care services likely played a role.^24^^,^^25^ This latter explanation is supported by quantitative evidence: a 2020 global survey of 337 audiologists reported reduced caseloads in 76.4% of respondents and shorter working hours in 38.7%, with in‑person core services such as hearing‑aid fitting and cochlear implant activation being particularly affected.^26^ Such disruptions may have led to substantial under‑diagnosis and under‑recording of cases during the early pandemic phase. As healthcare services resumed, these backlogged cases were identified in a concentrated fashion, and combined with the expanded surveillance scope of GBD 2023, this phenomenon may have manifested as a statistical rebound in DB. These interpretations remain speculative, however, and further empirical studies are needed to clarify the underlying drivers.

The heterogeneous correlations between PR, YLD, and SDI ‒ positive in high‑SDI regions and negative in middle‑ and low‑SDI regions ‒ can be explained by screening and diagnostic effects, as well as demographic structure. In high‑SDI areas, well‑established hearing screening and health statistics systems increase the detection rate of complete hearing loss, and prevalence of complete hearing loss accumulates with longer life expectancy. The opposite occurs in middle‑ and low‑SDI regions.^27^^,^^28^ Additionally, improved sanitary conditions reduce the burden of preventable deafness‑inducing factors in these regions.^27^

Globally and across the 21 GBD regions, the maximum possible DB reduction was not fully achieved in most or all years. Western Sub‑Saharan Africa, High‑income North America, High‑income Asia Pacific, North Africa and the Middle East, Southern Latin America, Southern Sub‑Saharan Africa, and Australasia have significant potential for improvement. These regions include four low‑ and middle‑income areas and three high‑income areas. In low‑ and middle‑income regions, limited public health resources and inadequate access to hearing aids hinder further DB reduction.^21^ A study of 665,000 individuals aged >50-years across 28 countries showed that hearing aid penetration was below 6% in high‑prevalence nations, including China, Mexico, and Brazil, whereas it reached 39% in Western and Northern Europe.^29^ Cochlear implantation is the preferred treatment for severe‑to‑profound hearing loss;^30^ however, awareness is low in some high‑income regions, and implantation rates therefore fall far below demand. In the US, only 10% of the population is aware of cochlear implants, and utilization rates range from 2% to 13%.^31^^,^^32^ In Australia, awareness is lacking among both professionals and the public, and the adult implantation rate is only 10%.^32^ In Japan, 75% of the population is unfamiliar with cochlear implants, and only 10%–20% of surgeries are performed in older adults.^33^ In less developed regions, services face structural scarcity and utilization barriers. Sub‑Saharan and West Africa have the lowest accessibility and a critical shortage of qualified staff, with limited human resources in ear and hearing care representing a major systemic challenge.^22^^,^^34^^,^^35^ In North Africa and the Middle East, the median time from diagnosis to implantation is 17-years.^36^ In southern Latin America, public insurance coverage varies widely; outcomes are better in younger patients, and assistive technologies, including implants, have long been neglected in research and practice.^37^^,^^38^

In High‑income North America, High‑income Asia Pacific, and Australasia, underutilized medical resources constitute the primary barrier to alleviating the DB among older adults with complete hearing loss aged ≥70 years. Only 25% of Australian adults have undergone hearing tests; merely 15%–30% of older hearing‑impaired Americans use hearing aids; and the penetration rate in Japan is approximately 14%.^39^, ^40^, ^41^ Institutional barriers further hinder service accessibility. For instance, hearing aids are not covered by Medicare in the United States, and over‑the‑counter devices lack professional fitting services.^42^ These factors account for the relatively heavy DB unexpectedly observed in High‑income North America and High‑income Asia Pacific.

From 2024 to 2040, the projected DB in high‑SDI regions shows a slight declining trend. The AR(1) coefficient revealed significant positive short‑term autocorrelation, suggesting temporal continuity in DB levels across adjacent years. The drift coefficient confirmed a statistically significant mild decreasing trend. The coexistence of positive short‑term dependence and negative long‑term drift suggests that the burden decline represents a systematic temporal variation. This modest decline implies diminishing marginal benefits of current interventions, highlighting the need for cost‑effective and innovative future strategies.

Annual variations globally and in the other four SDI regions are dominated by random fluctuations, with no statistically predictable systematic trends or stable extrapolatable patterns. Constant projected values do not equate to stable real‑world trends, nor do they indicate a burden plateau. Widening prediction intervals reflect growing uncertainty over time. Huang et al. predicted that complete hearing loss in most regions would slowly decline or fluctuate at a steady level rather than drop sharply, which is consistent with the weakened trend signals observed in our study.^43^

These factors may account for the ARIMA model prediction. Historical drivers of burden reduction ‒ such as infectious ear disease control and hearing screening ‒ have been nearly fully leveraged, whereas emerging factors, including accelerated population aging and improved access to hearing aids, have not yet formed stable long‑term trends. The GBD database continues to incorporate new data sources during version updates, particularly for previously data‑deficient, low‑resource areas. Such structural changes in the data foundation may alter the autocorrelation characteristics of the time series.

Previous studies fall into two main categories: those focusing on age‑related hearing loss, and those covering complete hearing loss across all age groups. The present study strictly restricted the study population to individuals aged ≥70 years with complete hearing loss, thereby filling the research gap concerning this older population with a heavy DB. Differences between our findings and prior studies stem from variations in population definition, age cutoff, and analytical methods.

This study offers novel clinical and public health insights. The declining DB and narrowed inequality provide quantitative evidence of improved global hearing health services, whereas the recent rebound reveals that health outcomes among older adults with complete hearing loss are highly vulnerable to external shocks. The seven priority intervention areas identified via frontier analysis can directly guide targeted allocation of global hearing health resources. Regional discrepancies in the correlation between DB and SDI indicate that mere comparison of PR may mislead resource allocation decisions. A comprehensive assessment incorporating population age structure, diagnostic capacity, and socioeconomic development is therefore indispensable. Given the uncertainty of burden projections, deterministic planning based on historical trend extrapolation should be replaced by dynamic monitoring and adaptive management. Measures include regular data updates, trend signal detection, and flexible adjustment of intervention strategies based on the latest evidence. High‑SDI regions can sustain improvements by leveraging their existing healthcare systems.

Based on our findings, the following countermeasures should be implemented. Clinically, it is necessary to consolidate consensus on active screening and early intervention, establish regular hearing screening programs for older adults, conduct periodic hearing assessments, and optimize the timing of clinical intervention. Remote fitting and basic rehabilitation services should be maintained during public health emergencies, such as pandemics. In public health practice, stabilizing DB without further deterioration is an acceptable goal under current intervention strategies. Future policies should shift focus from reducing prevalence to increasing rehabilitation coverage. Resources should be prioritized for the seven regions with the greatest improvement potential. In resource‑scarce areas, including Sub‑Saharan Africa, the burden can be efficiently reduced via task sharing, mobile screening, and affordable digital hearing aids. In developed regions such as High‑income North America and Asia‑Pacific, where hearing aid utilization is low, efforts should be made to raise public awareness, improve referral efficiency, and lower access barriers. Additionally, all regions need to establish hearing loss monitoring and early warning systems and integrate hearing health services into comprehensive geriatric assessment and emergency response plans for public health crises.

This study adopted a multidimensional and progressive strategy to systematically assess the epidemiological characteristics and future trends of complete hearing loss among the global population aged ≥70 years from 1990 to 2023. Specifically, EAPC and joinpoint regression models were applied to depict overall and phased trends and identify critical inflection points. Smoothing regression revealed the heterogeneous correlation between DB and SDI. Frontier analysis quantified the gap between actual regional burden and the theoretical optimum, extending the research scope from trend description to policy priority identification. Inequality analysis measured improvements in the equity of global hearing health services. The ARIMA time series model further expanded the research perspective from historical retrospective analysis to future projections.

This study has several limitations. First, inconsistent data sources across countries and regions may compromise the accuracy of the results. Second, confounding factors including evolving diagnostic criteria, regional disparities in healthcare accessibility and cultural differences were not incorporated, which may interfere with trend interpretation. Third, the ARIMA model lacked key covariates and was constrained by insufficient observations, limiting its capacity to capture complex and nonlinear variations. The predictions rest on the strong assumption that other factors remain unchanged; technological advances or abrupt demographic shifts may cause actual trends to deviate from projections. Systematic trends suitable for extrapolation cannot be identified based solely on historical data, resulting in limited predictive value. Fourth, this study did not conduct an in‑depth exploration of the diverse causes of complete hearing loss and their interaction with SDI, thus lacking evidence to support the formulation of targeted prevention and control strategies tailored to varying socioeconomic development levels and disease subtypes. Fifth, complete hearing loss among individuals aged ≥70 years primarily represents an irreversible chronic degenerative condition without a well‑defined onset point. Furthermore, the GBD 2023 database does not provide incidence data for this population; therefore, incidence was not incorporated in the present study. Sixth, incorporating Healthy Life Expectancy (HALE) would introduce substantial non‑hearing‑related confounding factors, reduce the specificity of relevant conclusions, and deviate from the core objective of assessing disease‑specific burden; therefore, HALE was not included.

## Conclusion

From 1990 to 2023, the overall DB of complete hearing loss among the global population aged ≥ 70 years experienced a slight decline. Inequality in DB showed moderate improvement across SDI regions, with generally favorable equity conditions. Seven GBD regions were identified as priority areas for subsequent hearing health interventions. The impact of uncertain future burden trends on health systems warrants ongoing attention. Regional disparities and the gap between actual burden reduction and theoretical optima in developed areas necessitate more targeted interventions. Additionally, it is crucial to enhance etiological monitoring systems and accurately document pathogenic factors.

## ORCID ID

Xiaoli Luo: 0000-0002-1861-3177

Di Wu: 0009-0000-5885-9131

Quanwei Ren: 0009-0002-6215-5027

Yilin Xiang: 0009-0003-6683-0904

## Funding

No financial support was received for the research or publication of this article.

## Declaration of competing interest

The authors declare no conflicts of interest.
